# Post-Silking Shading Stress Affects Leaf Nitrogen Metabolism of Spring Maize in Southern China

**DOI:** 10.3390/plants9020210

**Published:** 2020-02-06

**Authors:** Jue Wang, Kai Shi, Weiping Lu, Dalei Lu

**Affiliations:** 1 Jiangsu Key Laboratory of Crop Genetics and Physiology/Co-Innovation Center for Modern Production Technology of Grain Crops/Joint International Research Laboratory of Agriculture and Agri-Product Safety of the Ministry of Education of China, Yangzhou University, Yangzhou 225009, China; wj2847783561@163.com (J.W.); 005434@yzu.edu.cn (K.S.); wplu@yzu.edu.cn (W.L.); 2 Agricultural College, Yangzhou University, Yangzhou 225009, China

**Keywords:** shading, nitrogen metabolism, dry matter, grain yield, maize, SPAD

## Abstract

Lower sunlight caused by overcast skies from June to July in Southern China is one of the main environmental stresses that frequently occur and affect the post-silking growth and grain development of spring maize. In this study, a field trial involving four maize hybrids as materials was conducted to investigate the effects of post-silking shading stress (30% and 50% light deprivation) on leaf nitrogen metabolism and biomass accumulation during maize growing seasons in 2016 and 2017. Results indicated that 30% and 50% shading stress caused the grain yield to decrease by 47.3% and 69.6%, respectively. Plant post-silking biomass accumulation was decreased by shading, whereas the translocation from pre-silking assimilates in the vegetative organs was increased by shading. This change was sharply observed when the plants were deprived of more sunlight intensity. The leaf relative chlorophyll (soil and plant analyzer development (SPAD) value) and soluble protein contents were considerably decreased by shading under 50% light deprivation condition. The activities of nitrate reductase, glutamine synthetase and glutamate synthase that are involved in nitrogen metabolism were downregulated by shading stresses. In conclusion, nitrogen metabolism was disturbed by shading, which induced the decrease in post-silking dry matter accumulation, ultimately resulting in grain yield loss.

## 1. Introduction

Maize (*Zea mays* L.), a C_4_ crop, is the leading grain production cereal in the world, and its growth requires a high sunlight intensity [1]. In many regions in China, maize-growing regions are overcast in their later growth stages [2]. Shading during the whole growing season causes a decrease in the leaf area index, plant height and weights of stalk, cob and grain of maize [3]. Shading also decreases maize plant growth and delays development, causes tassel and ear infertility, reduces pollen vitality and silk differentiation and lowers kernel set percentage and grain dry matter accumulation, resulting in a reduced yield [4]. Shading at the flowering stage inhibits photosynthesis and diminishes kernel size, weight, glucose and starch contents [5]. Post-silking shading reduces the number of maize grains because of a limited source capacity [6], and a decreased kernel set is primarily in apical ear regions because of the decreased photosynthesis, the increased abscisic acid level and the nearly halted accumulation of nonstructural carbohydrate [7]. Plants suffering from a low sunlight intensity at the post-silking stage experience a dissolution of their cell membrane, karyotheca, mitochondria and some membrane structures, leading to a decreased photosynthetic capacity [8]. Root physiological properties (dry weight, root/shoot ratio, absorption area) are deteriorated by shading [9]. Additionally, leaf relative chlorophyll content and plant dry matter accumulation are reduced [10], resulting in grain yield loss. Under low light conditions, the activity of the carbon-concentrating mechanism generally decreases [1]. Shading at the kernel formation stage changes the maize leaf photosynthesis and chlorophyll fluorescence properties; it decreases the net photosynthetic and electron transport rates and increases the maximum and actual photochemical efficiency of Photosystem II (PSII) [11]. The decreased photosynthetic capacity may be due to the downregulated phosphoenolpyruvate carboxylase activity relative to Rubisco activity and to the lesser inhibition of nicotinamide adenine dinucleotide phosphate-malic enzyme relative to phosphoenolpyruvate carboxykinase, which perturbs the balance between the C_3_ and C_4_ cycles during photosynthesis in maize [12]. Post-silking low light after pollination decreases the volume of grain endosperm transfer cells and results in thin and short cell wall extensions of basal transfer cells, which leads to a decrease in maize grain weight [13]. The expression of microRNAs that regulate hormones, homeostasis, metabolism, development and flowering timing in maize ears is different between ambient light and shading treatments, resulting in the decreased maize yield [2].

Nitrogen (N) metabolism is an important substrate for energy metabolism that determines the yield and quality of crops. Plant photosynthetic capacity is closely associated with the leaf N status, such as leaf N content and N assimilatory enzyme activities [14]. The enzymes involved in assimilating intracellular ammonium into organic compounds are nitrate reductase (NR), glutamine synthetase (GS) and glutamate synthase (GOGAT). N metabolism is affected by reduced light in wheat, and a low GS and a high NR activity under low light conditions lead to photosynthesis and nitrate reduction [15]. Studies on cotton [16], grapevines [17] and tomatoes [18] observed that the activities of NR, GS and GOGAT in leaf tissues are reduced by shading. A study on strawberries [19] reported that the activity of NR in leaf tissues in response to shading was different among different NO_3_:NH_4_ ratios. However, limited studies have been performed to clarify the influence of light deprivation on leaf N metabolism in maize. In southern China, the climate from mid-June to mid-July is a plum rain season, and it induces a low sunlight intensity. That generates a detrimental effect on plant growth and development as it overlaps the grain filling stages of spring maize. Our former studies indicated that the volume and weight of grains and the starch deposition are suppressed [20,21], and starch quality is changed [22] by shading during grain filling. We hypothesized that downregulating the activities of leaf N metabolic enzymes may affect maize plant growth and grain development. In the present study, plants of four maize hybrids grown under ambient light, 30% and 50% light deprivation conditions were harvested, and the influence of shading on leaf N metabolism was clarified to help explain the causes of reduction in spring maize yield.

## 2. Materials and Methods

### 2.1. Plant Growth and Experimental Design

Four maize hybrids, namely, Zhengdan958 (ZD958), Jiangyu877 (JY877), Suyu30 (SY30) and Suyu29 (SY29), widely distributed in southeast China, were planted at Yangzhou University (32°39′74″N, 119°42′71″E), Yangzhou, China, in 2016 and 2017. The experimental soil was sandy loam with pH 6.8. The average contents of organic matter, alkali hydrolyzable N, Olsen-P, and exchangeable K in the plow layer (0–20 cm) were 13.2 g kg^−1^, 82.3 mg kg^−1^, 8.7 mg kg^−1^ and 73.9 mg kg^−1^ in 2016, and 14.7 g kg^−1^, 85.3 mg kg^−1^, 10.3 mg kg^−1^ and 82.2 mg kg^−1^ in 2017, respectively. The seeds were sown on 15 March and transplanted to the field on 28 March with a density of 75,000 plants ha^−1^ (60.0 cm × 22.2 cm) [22]. The plot size was 24 m^2^ (4 m × 6 m) with three replicates prepared in a randomized complete block design. The plants were initially treated with 600 kg ha^−1^ compound fertilizer (N/P_2_O_5_/K_2_O = 15%/15%/15%) at transplantation and followed by 500 kg ha^−1^ urea (N = 46%) at the eight-leaf stage.

At the silking stage, ears of 20 plants with similar progression levels were labeled, bagged and pollinated on the same day to reduce variation among plants. After manual pollination was completed, the plants were covered with a layer of black polyethylene nets that blocked approximately 30% (moderate shading, MS) and 50% (severe shading, SS) of solar radiation. The plants without shading were set as the control group (CK). The nets were placed at 450 cm above the ground to allow a good ventilation condition. The microclimate was determined by using a portable photosynthetic apparatus (LI-6400, LI-COR, Lincoln, Nebraska, USA) with a red–blue light source utilized to measure the related physiological parameters of each plant. The measurements were performed under the following conditions: an air temperature of 31 °C to 39 °C, a vapor pressure deficit of 0.5 to 1.0 kPa and an actinic light intensity of 1200 μmol m^−2^ s^−1^. Each measurement was performed at the center of the ear leaf and lasted approximately 2 min. The average light intensity, CO_2_, humidity and temperature at 10, 20, 30 and 40 days after pollination (DAP) were measured in each block.

### 2.2. Yield Determination

Two central lines were harvested to determine grain yield at maturity (harvest on 5 August and 6 August in 2016 and 2017, respectively), and the number of grains per ear was counted. The grains were manually stripped from the cobs and air dried, and the yield was calculated (kg ha^−1^). The harvested grain yield was determined at 14% moisture content [23].

### 2.3. Dry Matter Accumulation

Maize plants were sampled and separated into leaves, stems, sheaths and tassels once they reached the silking stage. At maturity, they were sampled and separated into leaves, stems, sheaths, tassels, cobs, husks and ears. The accumulation of dry matter and nutrients was limited to above ground because the root system was excluded [24]. All of the samples were oven dried at 80 °C to a constant weight after de-enzyming was conducted at 105 °C for 30 min.

The following equations were used for calculations: Post-silking dry matter accumulation (kg ha^−1^) = whole plant dry weight at maturity – whole plant dry weight at silking; Post-silking dry matter translocation (kg ha^−1^) = whole plant dry weight at silking – dry weight of vegetative organs (leaves, stems, sheaths and tassels) at maturity [25].

### 2.4. Relative Chlorophyll Content

The ear leaf relative chlorophyll content (SPAD value) was measured at 10, 20, 30 and 40 DAP in 10 randomly selected plants per treatment by using a portable chlorophyll meter (SPAD-502 Plus, Konica Minolta Inc., Tokyo, Japan).

### 2.5. Leaf Soluble Protein Content

After the midrib was removed, three ear leaves of each treatment at 10, 20, 30 and 40 DAP were cut in fragments, immediately frozen in liquid N_2_ and stored at −75 °C until the soluble protein content and the N metabolic enzyme activity were analyzed.

The soluble protein content in the ear leaves was estimated using the method proposed by Bradford [26], and bovine serum albumin was used as the standard.

### 2.6. Assay of NR, GS and GOGAT Activities

NR (E.C. 1.6.6.1) activity was determined in accordance with the method proposed by Majlath et al. [15]. GS (EC 6.3.1.2) and GOGAT (EC 1.4.1.13) activities were examined in accordance with the method developed by Liang et al. [27].

### 2.7. Statistical Analysis

Data reported in all figures and tables were expressed as the average of three repeated observations. Data were subjected to ANOVA with a least significant difference test at 5% probability level by using Data Processing System (7.05) [28].

## 3. Results

### 3.1. Post-Silking Weather

In Table 1, the temperatures and CO_2_ concentrations among the CK, MS and SS in both years were similar. The light intensity gradually decreased as the shading severity increased. The average values (means of 10, 20, 30 and 40 DAP) under CK, MS and SS were 1084.8, 704.0 and 458.5 μmol m^−2^ s^−1^ in 2016 and 1161.0, 762.8 and 632.5 μmol m^−2^ s^−1^ in 2017, respectively. The low light intensity in 2016 was due to intermittent rainfall during grain filling.

### 3.2. Grain Yield

**Figure 1:**
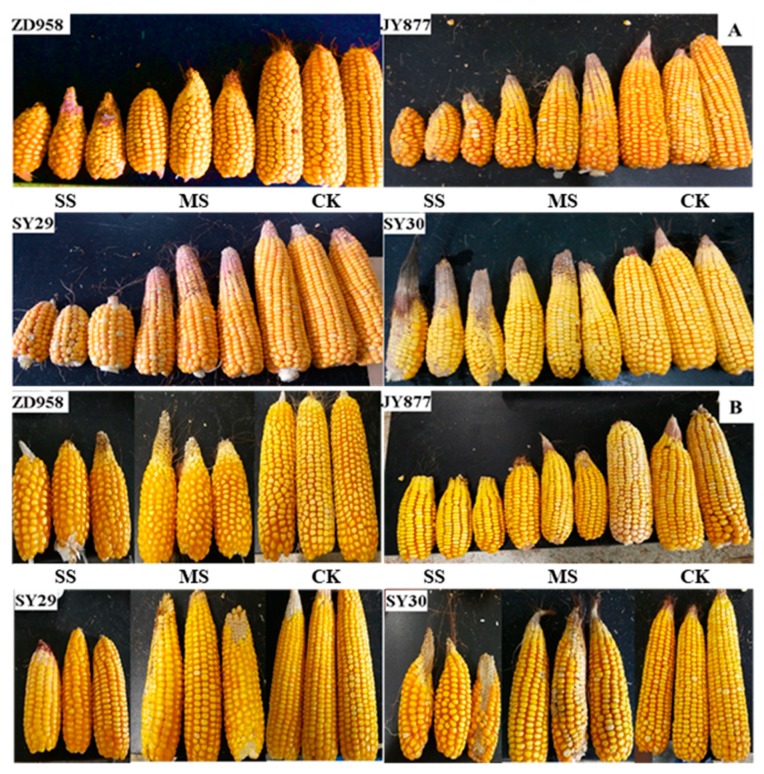
Typical morphology of ears for four maize hybrids under post-silking shading. CK, control; MS, moderate shading; SS, severe shading. A and B refer to 2016 and 2017, respectively. ZD958: Zhengdan958; JY877: Jiangyu877; SY29: Suyu29; SY30: Suyu30.

**Figure 2:**
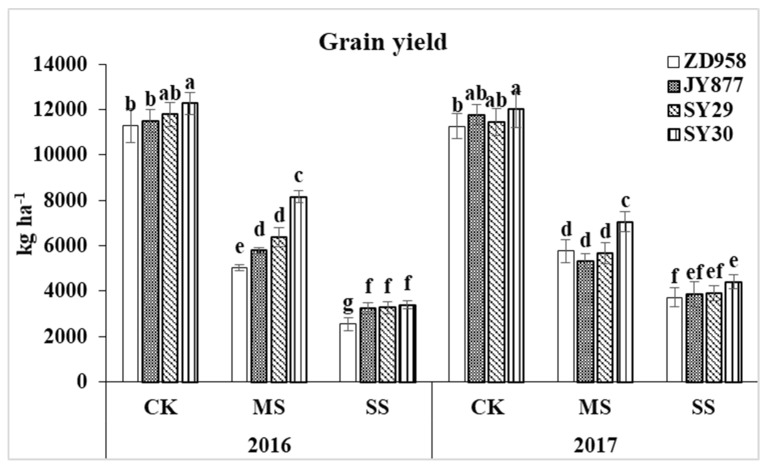
Grain yields of four maize hybrids under post-silking shading. CK, control; MS, moderate shading; SS, severe shading. ZD958: Zhengdan958; JY877: Jiangyu877; SY29: Suyu29; SY30: Suyu30. Values in the same year followed by different letters are significantly different (*p* < 0.05).

Post-silking shading decreased the ear size and grain numbers, leading to grain yield loss (Figure 1 and Figure 2), and the decrease was severe under SS. The average grain yields under MS and SS conditions were generally decreased by 45.9% and 73.4% in 2016 and by 48.8% and 65.8% in 2017, respectively. Amongst the four hybrids, SY30 had the highest grain yield under each condition (The average grain yields were 12,148, 7606 and 3900 kg ha^−1^ under CK, MS and SS treatments, respectively). This indicated that the grain yield potential of SY30 was higher than those of the three other hybrids.

### 3.3. Post-Silking Dry Matter Accumulation and Translocation

**Figure 3:**
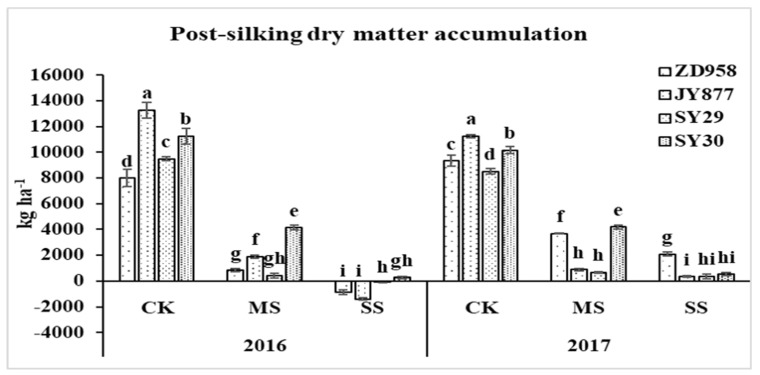
Plant dry matter accumulation of four maize hybrids under post-silking shading. CK, control; MS, moderate shading; SS, severe shading. ZD958: Zhengdan958; JY877: Jiangyu877; SY29: Suyu29; SY30: Suyu30. Values in the same year followed by different letters are significantly different (*p* < 0.05).

**Figure 4:**
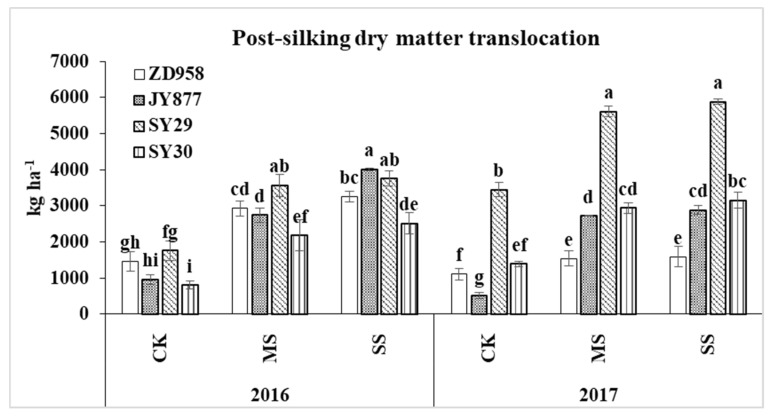
Plant dry matter translocation of four maize hybrids under post-silking shading. CK, control; MS, moderate shading; SS, severe shading. ZD958: Zhengdan958; JY877: Jiangyu877; SY29: Suyu29; SY30: Suyu30. Values in the same year followed by different letters are significantly different (*p* < 0.05).

The post-silking dry matter accumulation was the direct source of yield formation. Post-silking shading reduced dry matter accumulation (Figure 3). The biomass was reduced by 82.6% and 75.9% by MS in 2016 and 2017, respectively. Under the SS condition, the post-silking dry matter accumulation decreased by 91.5% in 2017, and the value was negative for all of the hybrids except SY30 in 2016, indicating that yield source was mainly dependent on the translocation of pre-silking assimilates stored in the vegetative organs. The dry matter translocation under MS and SS increased by 129.2% and 172.0% in 2016 and by 98.4% and 109.1% in 2017, respectively (Figure 4). Among the four hybrids, the average values of increase in the amount of translocation under MS and SS were 74.6% and 89.7% in ZD958, 271.6% and 368.9% in JY877, 75.9% and 84.9% in SY29 and 133.2% and 157.9% in SY30, respectively.

### 3.4. Leaf SPAD Value

**Figure 5:**
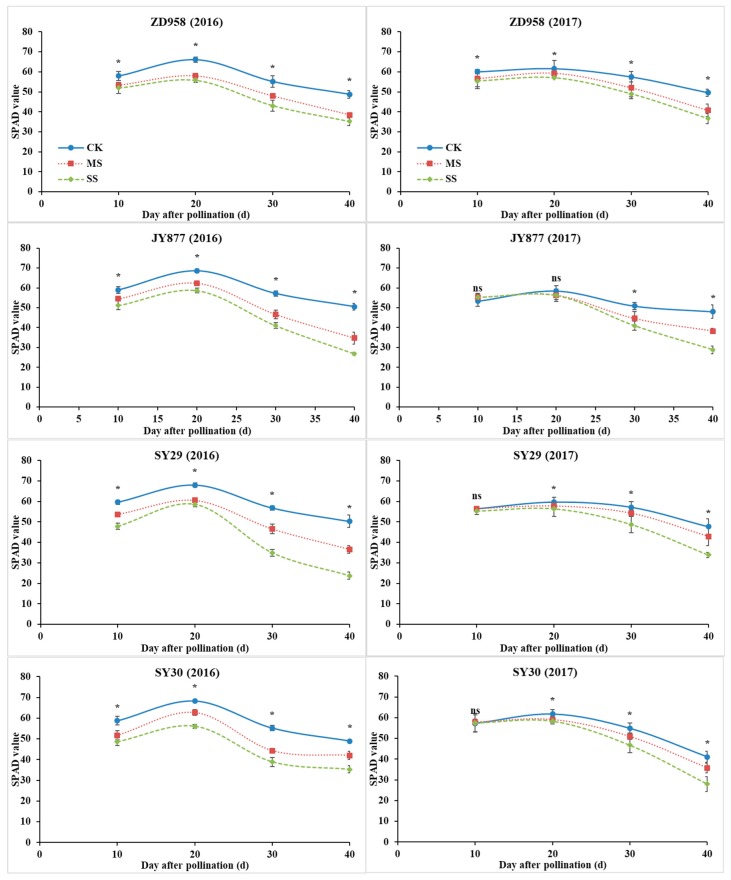
Ear leaf SPAD values of four maize hybrids under post-silking shading. CK, control; MS, moderate shading; SS, severe shading. ZD958: Zhengdan958; JY877: Jiangyu877; SY29: Suyu29; SY30: Suyu30. * means that the values at the same stage are significantly different (*p* < 0.05) among three treatments.

The leaf relative chlorophyll content (SPAD value) of the four hybrids increased initially, peaked at 20 DAP and decreased afterwards in both years. The SPAD value was decreased by shading, and the decrease was severe under SS in all hybrids in 2016 (Figure 5). In 2017, the SPAD value at 10 DAP only decreased in ZD958 and was unaffected in the other hybrids. The SPAD value at 20 DAP was unaffected in JY877, but the other three hybrids was decreased by shading, and the decrease was similar between MS and SS. At 30 and 40 DAP, the SPAD value was decreased by shading, and the decrease was severe under SS in all of the hybrids. In general, the average values under MS and SS were decreased by 14.5% and 23.8% in 2016 and by 6.5% and 12.7% in 2017, respectively. The following decreased values of the four hybrids under MS and SS were observed: 11.1% and 16.0% in ZD958, 11.7% and 18.2% in JY877, 10.1% and 20.9% in SY29 and 9.2% and 17.0% in SY30, respectively.

### 3.5. Leaf Soluble Protein Content

**Figure 6:**
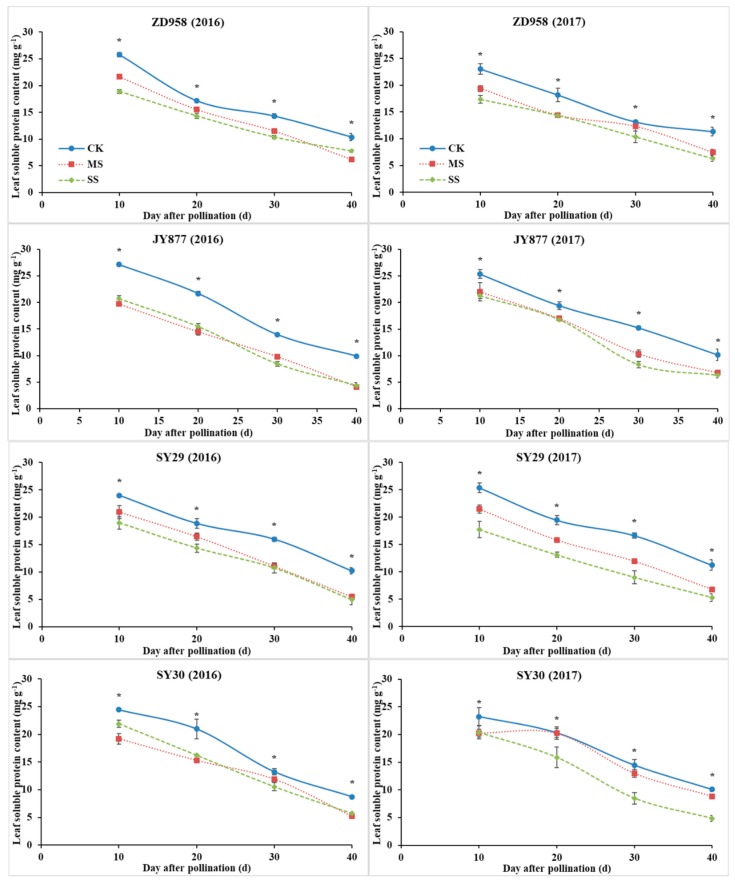
Ear leaf soluble protein contents of four maize hybrids under post-silking shading. CK, control; MS, moderate shading; SS, severe shading. ZD958: Zhengdan958; JY877: Jiangyu877; SY29: Suyu29; SY30: Suyu30. * means that the values at the same stage are significantly different (*p* < 0.05) among three treatments.

The leaf soluble protein content gradually decreased with grain growth. It was reduced by shading, but the reduction was dependent on year and hybrid (Figure 6). The value in ZD958 in 2016 was higher under MS than under SS before 30 DAP and lower under MS than under SS at 40 DAP. Conversely, the value in 2017 was higher under MS than under SS throughout the grain filling stage except at 20 DAP, and it was similar to that under SS. The value in JY877 was higher under MS than under SS at 30 DAP and was similar between the two shading treatments at the other stages. The value in SY29 in 2017 was higher under MS than under SS throughout the grain filling stage. Conversely, in 2016, the value was higher under MS before 20 DAP, and the difference disappeared thereafter. The value in SY30 in 2016 was higher under MS than under SS at 10 DAP and was similar between the two shading conditions thereafter. By contrast, the value in 2017 was similar between MS and SS at 10 DAP and higher under MS than under SS thereafter. In general, the soluble protein contents of ZD958, JY877, SY29 and SY30 decreased by 18.7%, 26.9%, 22.4% and 15.8% under MS and by 25.2%, 28.8%, 33.5% and 23.3% under SS, respectively.

### 3.6. GOGAT, GS and NR Activities

**Figure 7:**
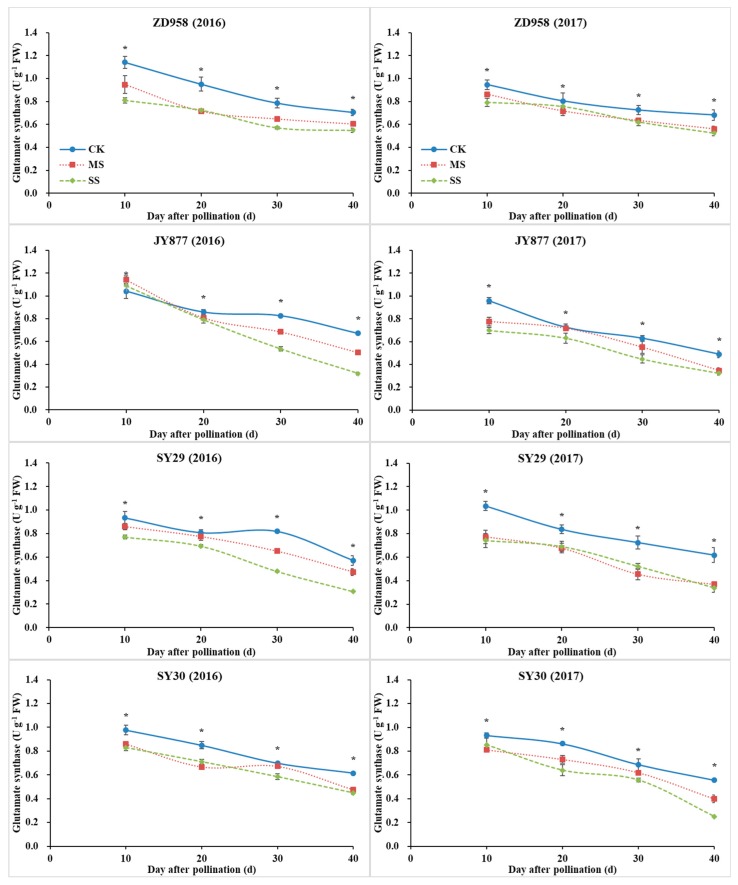
Ear leaf glutamate synthase (GOGAT) activities of four maize hybrids under post-silking shading. CK, control; MS, moderate shading; SS, severe shading. ZD958: Zhengdan958; JY877: Jiangyu877; SY29: Suyu29; SY30: Suyu30. * means that the values at the same stage are significantly different (*p* < 0.05) among three treatments.

**Figure 8:**
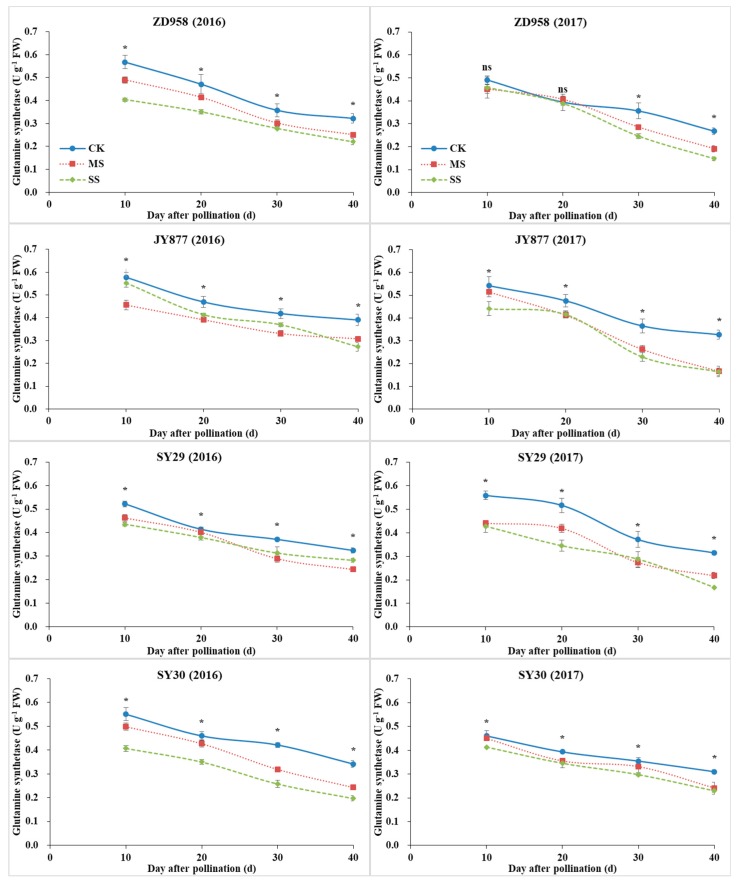
Ear leaf glutamine synthetase (GS) activities of four maize hybrids under post-silking shading. CK, control; MS, moderate shading; SS, severe shading. ZD958: Zhengdan958; JY877: Jiangyu877; SY29: Suyu29; SY30: Suyu30. * means that the values at the same stage are significantly different (*p* < 0.05) among three treatments.

**Figure 9:**
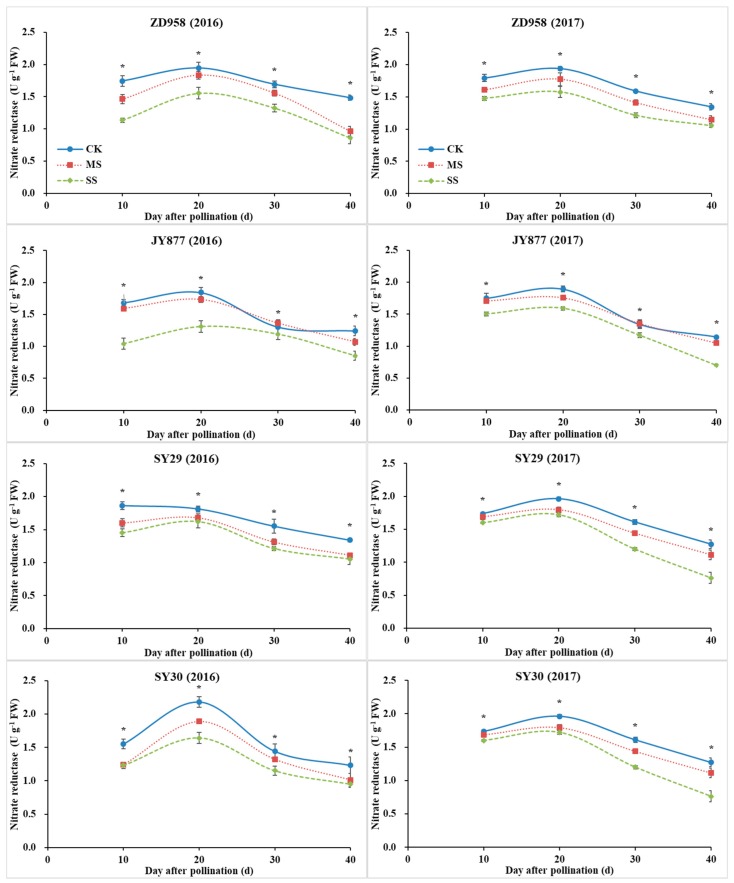
Ear leaf nitrate reductase (NR) of four maize hybrids under post-silking shading. CK, control; MS, moderate shading; SS, severe shading. ZD958: Zhengdan958; JY877: Jiangyu877; SY29: Suyu29; SY30: Suyu30. * means that the values at the same stage are significantly different (*p* < 0.05) among three treatments.

With plant development, the activities of GS and GOGAT gradually decreased, whereas the activity of NR increased initially, peaked at 20 DAP, and decreased afterwards (Figure 7, Figure 8 and Figure 9). The activities of GOGAT, GS and NR were decreased by shading, but the decrease was dependent on hybrid, stage and year. Compared with the control, the GOGAT activities of ZD958, JY877, SY29 and SY30 were decreased by 15.6%, 10.9%, 20.7% and 15.3% under MS and decreased by 20.8%, 22.1%, 28.4% and 21.1% under SS, respectively (Figure 7). Compared with the control, the GS activities of ZD958, JY877, SY29 and SY30 were decreased by 13.6%, 20.3%, 19.0% and 12.9% under MS and decreased by 22.8%, 19.9%, 22.3% and 24.0% under SS, respectively (Figure 8). Compared with the control, the NR activities of ZD958, JY877, SY29 and SY30 were decreased by 13.1%, 4.7%, 10.7% and 11.4% under MS and decreased by 24.7%, 23.2%, 19.3% and 21.0% under SS, respectively (Figure 9). Generally, compared with the control, the activities of GOGAT, GS and NR (mean of four hybrids at different stages) were decreased by 13.3% and 23.0%, 16.5% and 21.4%, and 12.2% and 24.5% under MS and SS in 2016 and decreased by 18.2% and 23.2%, 16.6% and 23.0%, and 8.0% and 19.7% in 2017, respectively.

## 4. Discussion

Maize grain yield is dependent on the post-silking photosynthate accumulation and the translocation of the reserved carbohydrates in vegetative organs, such as stems and leaves [29]. Post-silking direct assimilation is vital to grain development [30,31]. In the present study, shading decreased the post-silking direct dry matter accumulation, resulting in grain yield loss. The grain yield under shading was mainly dependent on the translocation of pre-silking reserved carbohydrate in stems and leaves. However, translocation could not compensate for the decreased post-silking biomass. The negative post-silking dry matter accumulation of ZD958, JY877 and SY29 in 2016 was possibly because of leaf and stem rotting caused by intermittent rainfall during grain filling. The reduced post-silking dry matter accumulation may be due to the decreased photosynthetic capacity attributed to light deprivation, which limits the source capacity for grain development [5,6,8,11]. The low SPAD value and photosynthetic rate (unpublished data) under shading indicated that the photosynthetic function deteriorated, and the leaf photoprotection mechanism was probably damaged, thereby decreasing photosynthesis and dry matter accumulation [10]. Nevertheless, studies on rice [32,33] and wheat [34] have found that leaf chlorophyll content is increased by shading, thereby improving the light harvesting potential to enhance light-use efficiency and reducing the dissipation of light energy. This discrepancy may be due to the difference between C_3_ and C_4_ crops. Xu et al. [35] also reported that slight shading delays wheat leaf senescence, enhances photosynthesis and grain filling and results in high grain yield, whereas mild and severe shading negatively affect grain yield.

The leaf N content and activity are closely associated with plant photosynthetic capacity. A high N content in leaves enhances photosynthesis and delays leaf senescence [36]. In the present study, the content of leaf soluble protein and the activities of NR, GS and GOGAT were reduced by shading, especially under severe shading treatments. A similar result is observed in cotton [16]. The leaf protein and chlorophyll contents under shading decrease as the photosynthetic rate and PSII photochemistry decrease and the peroxidation activity increases in wheat [37]. Setien et al. [38] observed that wheat behaves as a species sensitive to ammonium nutrition at a low light intensity, and the low GS activity is insufficient for ammonium assimilation. However, a study on wheat has observed that the activity of NR is unaffected by shading at ambient temperatures, and its value increases at low temperatures [15]. Thus, the increased activity of NR at a low temperature might help sustain nitrate reduction.

## 5. Conclusions

The leaf relative chlorophyll content, soluble protein content and NR, GS and GOGAT activities were reduced by post-silking light stress. These results indicated that the leaf pigments and N metabolism were affected by shading, and the translocation of pre-silking assimilates that stored in vegetative organs could not compensate for the reduction of post-silking biomass accumulation, leading to grain yield loss. Therefore, in southern China spring maize production, selecting low-sunlight tolerant hybrids, adjusting nitrogen applications or spraying exogenous hormones to enhance leaf nitrogen metabolism and keep a high chlorophyll content could increase the dry matter accumulation and alleviate the negative influence of weak-light stress.

## Figures and Tables

**Table 1:** Environmental conditions during grain filling under different treatments.

Treatment	Air Temperature (°C)		Relative Humidity (%)		CO_2_ Concentration (µmol mol^−1^)		Light Intensity (µmol m^−2^ s^−1^)	
	2016	2017	2016	2017	2016	2017	2016	2017
CK	34.3	36.0	58.2	60.0	382.8	384.5	1084.8 a	1161.0 a
MS	34.2	35.9	58.2	59.3	390.0	386.3	704.0 b	762.8 b
SS	34.3	35.9	57.2	59.6	392.8	391.8	458.5 c	632.5 c

Within column, numbers followed by different letters indicate significant differences (*p* < 0.05). CK, control; MS, moderate shading; SS, severe shading.
